# Determinants of morbidities and mortality in acromegaly

**DOI:** 10.20945/2359-3997000000193

**Published:** 2019-11-01

**Authors:** Leandro Kasuki, Paula da Silva Rocha, Elisa Baranski Lamback, Mônica Roberto Gadelha

**Affiliations:** 1 Faculdade de Medicina Hospital Universitário Clementino Fraga Filho Universidade Federal do Rio de Janeiro Rio de Janeiro RJ Brasil Centro de Pesquisa em Neuroendocrinologia, Divisão de Endocrinologia, Faculdade de Medicina e Hospital Universitário Clementino Fraga Filho, Universidade Federal do Rio de Janeiro, Rio de Janeiro, RJ, Brasil; 2 Serviço de Neuroendocrinologia Instituto Estadual do Cérebro Paulo Niemeyer Rio de Janeiro RJ Brasil Serviço de Neuroendocrinologia, Instituto Estadual do Cérebro Paulo Niemeyer, Rio de Janeiro, RJ, Brasil; 3 Serviço de Endocrinologia Hospital Federal de Bonsucesso Rio de Janeiro RJ Brasil Serviço de Endocrinologia, Hospital Federal de Bonsucesso, Rio de Janeiro, RJ, Brasil; 4 Laboratório de Neuropatologia e Genética Molecular Instituto Estadual do Cérebro Paulo Niemeyer Rio de Janeiro RJ Brasil Laboratório de Neuropatologia e Genética Molecular, Instituto Estadual do Cérebro Paulo Niemeyer, Rio de Janeiro, RJ, Brasil

**Keywords:** Acromegaly, systemic complications, mortality, cardiovascular disease, cancer

## Abstract

Acromegaly is a systemic disease associated with increased morbidity, presenting cardiovascular, metabolic, respiratory, neoplastic, endocrine, articular and bone complications. Most of these comorbidities can be prevented or delayed with adequate disease treatment and, more recent studies with the use of modern treatments of acromegaly, have shown a change in the severity and prevalence of these complications. In addition, acromegaly is associated with increased mortality, but recent studies (especially those published in the last decade) have shown a different scenario than older studies, with mortality no longer being increased in adequately controlled patients and a change in the main cause of death from cardiovascular disease to malignancy. In this review, we discuss this changing face of acromegaly summarizing current knowledge and evidence on morbimortality of the disease. Arch Endocrinol Metab. 2019;63(6):630-7

## INTRODUCTION

Acromegaly is a rare disease characterized by excessive growth hormone (GH) and increased insulin-like growth factor I (IGF-I), which is, in the vast majority of cases, caused by GH-secreting pituitary adenoma (1). It is associated with increased morbidity due to secondary systemic complications that include cardiovascular, cerebrovascular, respiratory, osteoarticular systems, as well as endocrine and metabolic alterations and neoplasias (1). Mortality rate is also increased in acromegaly and quality of life is decreased (1).

## ACROMEGALY COMORBIDITIES

### Metabolic complications

**Figure 1:**
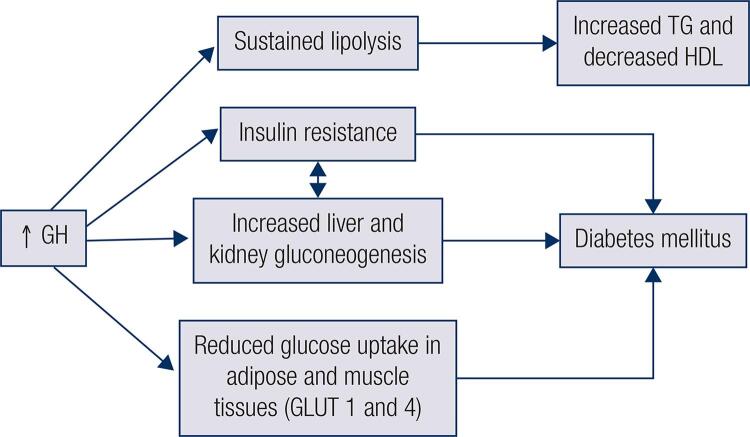
Effects of acromegaly on glucose and lipid metabolism. GH: growth hormone; GLUT: glucose transporter; HDL: high-density lipoprotein; TG: triglycerides.

Patients with acromegaly have metabolic complications that affect both glycemic and lipid metabolism and is mostly due to GH excess (2). Most patients have insulin resistance (IR) with impaired insulin sensitivity, and increased liver and kidney gluconeogenesis due to chronic GH excess, which contributes to glycemic abnormalities (3) (Figure 1). During fasting, GH is the major anabolic hormone counteracting insulin and in excess leads to sustained stimulation of lipolysis and lipid oxidation (2). Acromegaly effects on glucose metabolism are mainly caused by insulin-antagonic effects of chronic GH excess (4). GH inhibits lipoprotein lipase activity in adipose tissues, leading to increase in the efflux of free fatty acids (FFA) to the liver, which in turn favors IR, increases the synthesis of triglycerides, reduces high-density lipoprotein (HDL) levels and body fat (2) (Figure 1). This sustained lipolysis contributes to acromegaly’s unique metabolic changes in which IR can be seen along with reduced body fat. Moreover, reduced glucose uptake in adipose tissue and muscle with lower expression of glucose transporter-1 and 4 are also observed in acromegaly (2,3) (Figure 1). In contrast, IGF-I has opposing actions compared to GH under physiological conditions. It promotes FFA uptake into adipose and liver tissues, leading to reduced FFA, as well as increased glucose uptake and insulin sensitivity primarily on skeletal muscles (5). However, in acromegaly, IR predominates and IGF-I’s potentially beneficial effects are counteracted. This leads to higher prevalence of glucose and lipid abnormalities in acromegaly patients compared to normal population (6).

In newly diagnosed acromegaly patients, about 50% of patients develop some type of glucose impairment characterized by altered fasting plasma glucose, impaired glucose tolerance or diabetes mellitus (DM) (7). With regards to DM alone, it is present in approximately 30% of patients, but has been described in up to 56% of patients, depending on the presence of other risk factors (1,7). Severity of glucose abnormalities is related to positive family history of DM, increased body mass index (BMI), older age, as well as GH and IGF-I levels (3).

Hyperlipidemia is also present in up to 50% of patients and is mainly characterized by hypertriglyceridemia and reduced HDL levels (1,6). Although concentrations of low-density lipoprotein (LDL) have been shown to be increased or similar to normal subjects, higher levels of oxidized LDL have been described (6,8,9). Taken together, IR is the main metabolic abnormality seen in acromegaly patients (4).

### Cardiovascular disease

**Figure 2:**
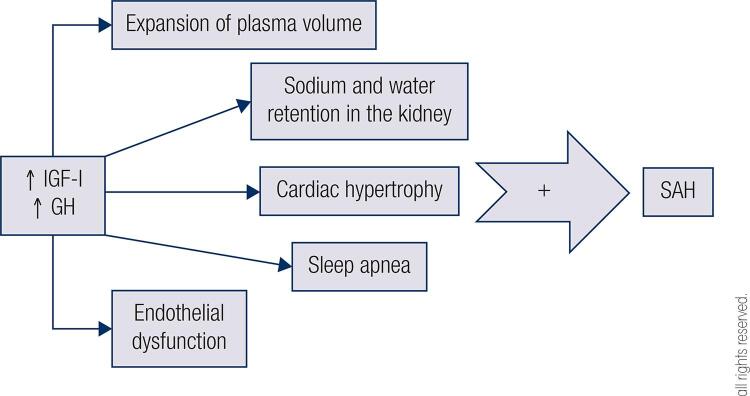
Effects of acromegaly in systemic arterial hypertension. SAH: systemic arterial hypertension; GH: growth hormone; IGF-I: insulin-like growth factor I.

Cardiovascular disease is one of the most prevalent comorbidities in patients with acromegaly, with arterial hypertension being the most common disorder, with prevalence ranging from 18% to 60% and being present since early stages (1,10). It is characterized by elevated diastolic blood pressure and higher prevalence of non-dippers (11). No relation was found with gender or family history of hypertension, but there is an association with higher IGF-I levels demonstrated in some studies and with the duration of GH hypersecretion (1,11). The pathogenic mechanisms of arterial hypertension are multifactorial and the most accepted cause is the expansion of plasma volume and sodium and water retention in the kidney (1) (Figure 2). Other conditions, such as cardiac hypertrophy and/or sleep apnea, may contribute to the increase in blood pressure levels (Figure 2). With prolonged acromegaly duration there are secondary changes in the vascular system and cardiac remodeling that also help exacerbate arterial hypertension (12). Prevalence of arterial hypertension may be overestimated by office measurement of blood pressure, as lower prevalence is observed when 24-hour ambulatorial blood pressure monitoring is used (13).

**Figure 3:**
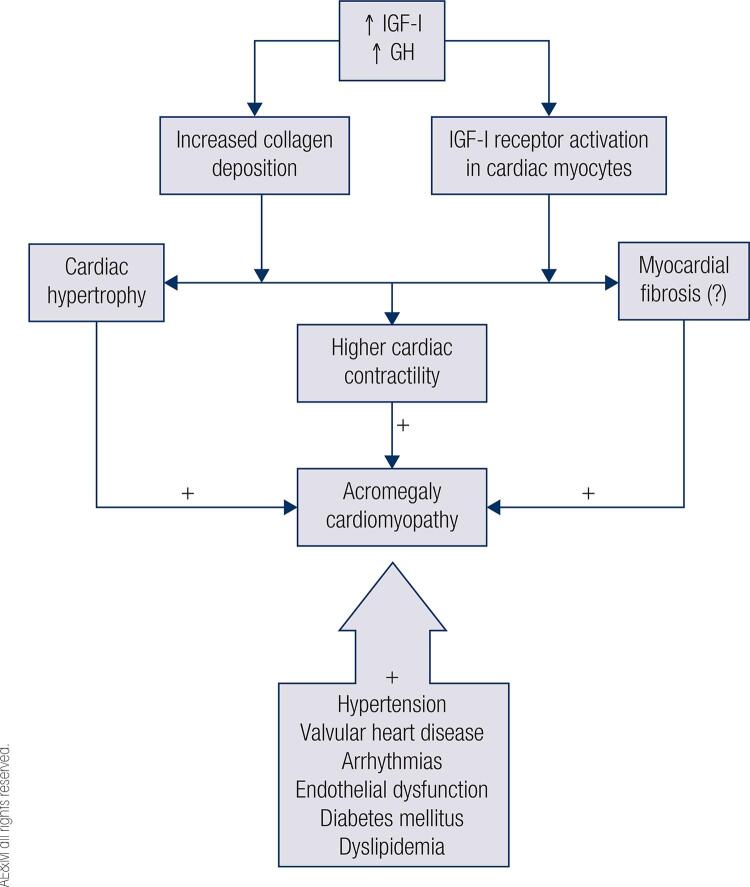
Acromegaly cardiomyopathy. GH: growth hormone; IGF-I: insulin-like growth factor I.

Acromegaly cardiomyopathy, another frequent complication of GH hypersecretion, is characterized by concentric biventricular hypertrophy, diastolic dysfunction and mitral and aortic valve disease (1,14). Myocardial hypertrophy may occur in the early stages of acromegaly and has worse evolution with maintenance of GH and IGF-I excess (12). These stimulate collagen deposition, increase IGF-I receptor activation in cardiac myocytes that will result in higher cardiac contractility, cardiac hypertrophy and possible myocardial fibrosis, although studies are discordant regarding the latter (15) (Figure 3). The prevalence of left ventricular hypertrophy (LVH) ranges from 11% to 78% in echocardiographic studies (1). More recently, the prevalence of LVH has been studied by cardiac magnetic resonance imaging, but results still differ regarding the actual prevalence of LVH, with the largest series in the literature, from our group, showing a similar prevalence of myocardial fibrosis in patients with acromegaly compared to the healthy population and a lower frequency of LVH (5%) (16).

The severity of heart disease may also be due to the existence of some comorbidities such as arterial hypertension, valvular heart disease, arrhythmias in addition to endothelial dysfunction itself, DM and dyslipidemia (12). Long-term (6 to 24 months) treatment of acromegaly with first-generation somatostatin receptor ligands (SRL) improves cardiomyopathy, reduces LVH and may improve diastolic dysfunction (1).

Despite many studies showing LVH and diastolic dysfunction as common features of acromegaly, evolution to systolic dysfunction is uncommon (1). The majority of studies published in the last 15 years, reflecting contemporary cohorts treated with more modern therapeutic armamentarium show lack of systolic dysfunction or presence of this complication in less than 3% of the patients with our group showing no difference in left ventricular ejection fraction between acromegaly patients and healthy patients (66.9% versus 68.4%) (1,14).

Acromegaly typically affects mitral and/or aortic valves, presenting a prevalence of valvular cardiac disease of up to 75% at diagnosis (11). Valvulopathy is a consequence of collagen and mucopolysaccharides deposition in valvular leaflets and deregulation of extracellular matrix that result in ring fragility and valve regurgitation (1). The risk factors for valve disease are presence of arterial hypertension and duration of acromegaly, unrelated to GH or IGF-I levels in some studies, although a prospective study shows this relation to mitral regurgitation (1). Our group described for the first time the occurrence of aortic ectasia that have been seen in up to 26% of patients with acromegaly and is related to the increased frequency of aortic regurgitation (1,10).

Another cardiovascular complication found in patients with acromegaly is abnormalities of cardiac rhythm that are observed in 7 to 40% of these patients, and are more frequent during exercise (12). However, recent studies with 24-hour Holter, including one from our group, showed no clinically significant or sustained arrhythmias in patients with acromegaly (1,17). Different types of arrhythmias are described in acromegaly including paroxysmal atrial fibrillation and supraventricular tachycardia, sick sinus syndrome, isolated and paired ventricular ectopic beats, and ventricular tachycardia (11). In recent cohort studies, some possible mechanisms have been described including a longer QT duration or dispersion, higher frequency of late potentials and reduced normal-to-normal heart period (1).

Patients with acromegaly may also have a left ventricular dyssyncronicity that consists of loss of the simultaneous peak contraction of corresponding cardiac segments (11). This peculiar rhythm abnormality is independent of typical cardiovascular disease risk factors and a direct effect of hormonal excess on cardiac synchronicity is suggested (18).

The prevalence of atherosclerosis in acromegaly is controversial (1). Some studies show higher prevalence, but the majority of studies show either equal prevalence or even lower prevalence than normal population (1). Some reasons for this discrepancy are the heterogeneity of diagnostic methods used for its definition and the influence of age, gender and presence of concomitant cardiovascular risk factors (smoking, sleep apnea, IR, hyperglycemia, arterial hypertension, dyslipidemia, and overweight) (12). Chronic hormonal excess does not appear to contribute to atherosclerosis directly (18). Coronary heart disease is more related to arterial hypertension, DM and dyslipidemia and does not seem to be increased in patients with acromegaly (1).

### Cerebrovascular disease

Acromegaly has higher incidence of arterial hypertension, IR and DM that predispose to cerebrovascular events (19). Thus, stroke prevalence is expected to be higher in patients with acromegaly. However, the incidence of stroke is not higher compared with the general population, showing great relevance in comorbidities rather than acromegaly *per se*. (1). This scenario changes when radiotherapy is used, increasing the incidence of stroke and its mortality (1). These alterations seem to be a direct effect of radiotherapy, as incidence of cerebrovascular disease also increases in other non-secreting GH tumors undergoing radiotherapy (1).

### Respiratory disease

**Figure 4:**
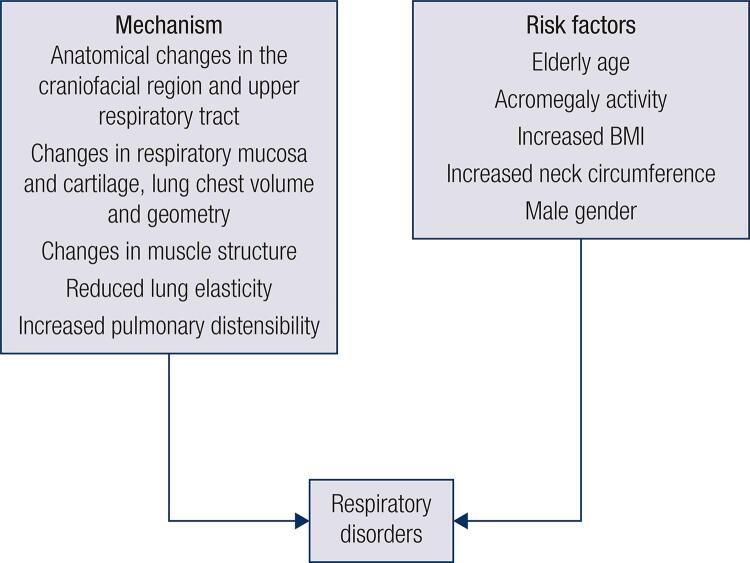
Respiratory disorders in acromegaly. BMI: body mass index.

Respiratory disorders are common in acromegaly and occur due to anatomical changes in the craniofacial region and upper respiratory tract such as tongue swelling, changes in respiratory mucosa and cartilage, lung chest volume and geometry, along with changes in muscle structure, reduced lung elasticity, and increased pulmonary distensibility (18,20) (Figure 4).

The most frequent respiratory disease is sleep apnea syndrome (SAS), being obstructive SAS (OSA) the main type (20). Obstructive sleep apnea syndrome is present in high frequency in patients with acromegaly (95.3%) and is more common in patients with higher levels of GH and IGF-I (1,20). Potential risks for respiratory disease are elderly age, acromegaly activity, increased BMI and neck circumference, and male gender, which are associated with two to three times more likelihood to have apnea (18) (Figure 4). Another type of SAS is central sleep apnea characterized by cessation of brain control of respiration (18). This disorder has been found in one third of patients with acromegaly and may be directly related to high GH or IGF-I levels (1). Acromegaly treatment most often improves SAS, but biochemical control does not necessarily reverse the obstructive disease (1).

Respiratory insufficiency, a poorly investigated comorbidity in acromegaly, affects 30% to 80% of patients (18). About 70% of patients with acromegaly achieve lower respiratory muscle strength on both inspiration and expiration (18). The pathogenesis of this disorder seems to be due to the narrowing of small and upper airways and increase in lung volume and lung capacity (18). However, a significant correlation between lung volume and upper airway narrowing in relation to high GH and IGF-I levels has not been proven (1). Acromegaly patients have non-aerated and poorly ventilated areas in their lungs, which leads to poor ventilation (18). Duration of inspiration is generally shorter and respiratory rate may be increased. Subclinical hypoxemia may be seen in up to 80% of patients due to poor ventilation-perfusion ratio, nocturnal hypoxemia that is reported in 23% and an inadequate response to ventilatory demand during exercise (18). The potential benefit of acromegaly control in respiratory function has yet to be elucidated (1).

### Neoplastic complications

Overall, the true prevalence and risk of malignancy in acromegaly remains unknown, but risk does not seem to be increased for the majority of cancers (1). Epidemiological studies show that acromegaly patients are at increased risk of developing benign and malignant tumors arising from the colon and thyroid gland (1,21,22). Also, its physiopathology is controversial. The exact mechanism of tumorigenesis is unknown. Increased GH and IGF-I secretion in a prolonged manner are thought to promote tumor development and progression because of cellular growth stimulation and cellular proliferation (4). IGF-I has mitogenic and anti-apoptotic activity (1). Proto-oncogene expression are also possibly involved (23). Nevertheless, there is no evidence for enhanced *de novo* tumorigenesis in acromegaly (1). Evidence from other studies in non-acromegaly populations suggests that IR and metabolic syndrome are associated with increased cancer incidence (24).

Compared to general population, acromegaly patients have 2.4-fold increased risk of colonic adenomas and a 7.4-fold greater risk of cancer (25,26). The majority of colon cancers develop from malignant transformation of benign adenomatous colonic polyps (27). Acromegaly patients usually are diagnosed with a gap of 10 years, potentially resulting in the development of pre-existing colonic tumors, or initiation of their development (27). Furthermore, colon cancer is also associated with modifiable factors, such as diet, obesity, DM, hypertriglyceridemia and physical inactivity, that are related to hyperinsulinemia and RI, which may play a role in the colorectal tumorigenesis and also to non-modifiable factors, such as genetic and epigenetic mechanisms (28).

With regard to thyroid gland, nodular thyroid disease is frequently seen in patients with acromegaly and has been associated with acromegaly disease duration, GH and IGF-I levels (1,29). Up to 75% of patients with acromegaly have nodular thyroid goiter with similar incidence among genders, different from general population (30,31). Regarding thyroid cancer, acromegaly patients have 4.1-fold increased risk of thyroid cancer compared to case-control, but this risk may be overestimated (1). It has no association with acromegaly disease activity or duration, and has similar natural history compared to general population, with papillary thyroid cancer being the most frequent type (1,32).

With respect to other cancers such as breast, prostate and kidney, no increase in risk has been conclusively observed, although GH signal transduction pathway and RI are key determinants associated with breast cancer susceptibility (33,34).

### Joint and bone complications

Joint complications and fractures are more common in acromegaly patients than in the general population (35,36). Arthropathy is present in 20 to 70% of patients, affecting weight- and non-weight-bearing joints, particularly shoulders, knees and hips (37). Its prevalence and severity seems to be associated with higher baseline GH and IGF-I levels at diagnosis and duration of uncontrolled disease (37,38). In almost all patients, radiological manifestations of osteoarthritis in at least one joint site is seen, mainly spine and hip (1,39). Its pathophysiology is similar to that of primary osteoarthritis, being considered degenerative, with one difference: presence of cartilage hypertrophy with severe osteophytosis, resulting in joint space widening, rather than narrowing due to cartilage loss (39). Acromegaly arthropathy significantly impact on patients’ quality of life (40).

With respect to bone complications, patients with acromegaly exhibit increased bone remodeling caused by excess GH leading to deterioration of bone microarchitecture and impairment of bone strength (41,42). Evidence for increased bone turnover has been shown in histomorphometry studies with increased markers of bone reabsorption compared to bone formation (43,44). Acromegaly is known to be a cause of secondary osteoporosis and is associated with increased risk of vertebral fractures (VF) (45). Up to 60% of patients present with radiological VF, which is independent of bone mineral density (36,46). It correlates with disease activity and duration, although it also occurs in patients with biochemical control (about 25% of patients) (36,46). Fractures are most frequent in the thoracic spine in which lower trabecular bone is seen and occurs as early as 2-3 years after diagnosis (36,46). The risk of fractures is also increased in hypogonadic patients and in the presence of DM (36,47).

Moreover, hypercalcemia and hypercalciuria have been reported in about 10% and up to 70% of acromegaly cases, respectively, contributing to the increased frequency of nephrolithiasis in acromegaly patients, and is associated with disease activity (43,48). These findings can be seen due to increased 1,25(OH)_2_ vitamin D from renal activation of 1-alpha hydroxylase by GH resulting in increased intestinal absorption of calcium or, less frequently, due to concomitant primary hyperparathyroidism (49). GH excess contributes to increased bone turnover leading to hypercalciuria (50). Patients with active acromegaly also have higher phosphate levels because of direct antiphosphaturic action of IGF-I in the proximal tubule (50). Hypercalciuria and phosphate levels can be considered as markers of skeletal fragility and disease activity (50).

### Endocrine complications

Hyperprolactinemia can be seen in acromegaly either due to tumor co-secretion (approximately 25% of somatotropinomas may co-secrete prolactin) or to stalk compression (with consequent reduction of the dopaminergic tone), leading to hypogonadism (1).

In addition to hypogonadism secondary to hyperprolactinemia, acromegaly patients may present pituitary deficits secondary to compression of normal pituitary or pituitary stalk by the somatotropinoma (1,51). Additionally, hypopituitarism can be a consequence of acromegaly treatment, especially radiotherapy and this is illustrated by the reduction of the frequency of hypopituitarism in acromegaly patients in more recent series (around 25%) in comparison to older series (around 40%), in which there was more use of radiotherapy (1,35,52).

### MORTALITY IN ACROMEGALY

Acromegaly is associated with increased mortality as a consequence of its main comorbidities reported above, but this increased mortality can be reverted by adequate treatment leading to disease control (1,53). In older studies, published before 1995, this mortality ratio was twice or thrice that of normal population (30,54). Two posterior meta-analysis published in 2008, showed that, at that time, mortality was still increased in active disease patients, but it was about 1.7 that of normal population (55,56). However, the recent advance in acromegaly treatment and in the treatment of its comorbidities, has led to normalization of mortality in adequately treated patients, as well as a change in the main cause of death in acromegaly (1). This is well illustrated in the largest series in the literature, an Italian multi-center study including 1512 patients (57). In this study, disease control was observed in 65% of patients and standardized mortality ratio (SMR) was not increased [1.13 – 95% confidence interval (CI) 0.87 – 1.46]. Mortality was only higher in those patients with active disease [SMR 1.93 (95% CI 1.34 – 2.70)] (57).

The reduction of acromegaly mortality was also well illustrated in the most recent meta-analysis published in 2018 (53). In the 17 studies published before 2008, mortality was increased [SMR 1.76 (95% CI 1.52 – 2.40)], while in the nine studies published after 2008, mortality was equal to normal population [SMR 1.35 (95% CI 0.99 – 1.85)] (53).

### Determinants of mortality in acromegaly

Two main factors seem to impact the mortality rate: disease activity and treatment modality applied to achieve disease control (1,53).

In an interesting study by Colao and cols. (58) two different cohorts from Bulgaria (n = 407) and Italy (n = 220) were compared. They differed mainly in the treatment modalities, with a higher frequency of treatment with SRL and pegvisomant in Italy, resulting in a lower rate of radiotherapy treatment. Additionally, disease control was more frequent in the Italian cohort than in the Bulgarian series (50% vs 39%, respectively, p = 0.005) at last follow-up. As a result, Bulgarian cohort had a higher mortality than normal population [SMR 2.0 (95% CI 1.54 – 2.47)], while the Italian cohort had a normal mortality rate [SMR 0.66 (95% CI 0.27 – 1.36)]. Interestingly, in the Bulgarian cohort, those patients who achieved disease control had a normal SMR [1.25 (95% CI 0.68 – 1.81)]. In this study, age at diagnosis and last GH value were related to all-cause mortality, while radiotherapy was associated with cerebrovascular mortality (58).

Other studies have shown different factors impacting mortality in acromegaly (51,57). In the previously cited largest literature series, older age, GH levels at last follow-up, IGF-I levels at diagnosis, malignancy and radiotherapy were independent predictors of mortality (57). Another important consequence of acromegaly *per se* or of some of its treatment (surgery and mainly radiotherapy), hypopituitarism, is associated with increased mortality (1,51). Main determinant seems to be ACTH deficiency, being associated with a SMR of 1.7 (95% CI 1.2 – 2.5) after multivariate analysis in the study by Sherlock and cols. (51).

### Causes of death

In an extensive review published in 2004, Colao and cols. (30) showed that at that time, mortality was increased in acromegaly and was mainly caused by cardiovascular disease (60%), followed by respiratory disease (25%), with malignancy being responsible for only 15% of the cases. As previously reported, more recent studies, especially those published in the last decade have shown a change in the mortality ratio in acromegaly and this was accompanied by a change in the disease face, with cardiovascular disease being no long the main cause of death (1).

The majority of studies published from 2004 to 2019 have shown malignancy as the main cause of death in acromegaly (1). This was the case of four out of the seven studies published in the last decade (35,57,59,60). The most recent meta-analysis that analyzed mortality in acromegaly corroborated these data, showing that cancer was the main cause of death in the studies published in the last decade, coinciding with a higher life expectancy in this population (53).

## CONCLUSIONS

Acromegaly is a chronic systemic disease, associated with many complications in the presence of active disease. In the last decades, significant advance in acromegaly treatment and also in the treatment of its comorbidities has changed the face of disease, with reduction of frequency of some complications, like cardiovascular disease, and also resulting in normalization of mortality on those patients adequately treated (1,53). Lastly, main cause of death has changed from cardiovascular disease to cancer in contemporary cohorts (1).
